# Supplementary material for: HTRA1 disaggregates α-synuclein amyloid fibrils and converts them into non-toxic and seeding incompetent species
Source: Nat Commun. 2024 Mar 18;15:2436. doi: 10.1038/s41467-024-46538-8 (PMC10948756; doi:10.1038/s41467-024-46538-8)
Supplement: Supplementary file 1 — Supplementary Information [file 41467_2024_46538_MOESM1_ESM.pdf]

Supplementary Information

HTRA1 prevents and reverses α-synuclein aggregation, rendering it non-toxic and seeding incompetent

Sheng Chen[1], Anuradhika Puri[1], Braxton Bell[1], Joseph Fritsche[1], Hector H. Palacios[1], Maurie Balch[1], Macy L. Sprunger[1], Matthew K. Howard[1], Jeremy J. Ryan[1], Jessica N. Haines[2], Gary J. Patti[1,3], Albert A. Davis[2], and Meredith E. Jackrel[1*]

[1]Department of Chemistry, Washington University, St. Louis, MO 63130, U.S.A.
[2]Department of Neurology, Washington University, St. Louis, MO 63130, U.S.A.
[3]Department of Medicine, Washington University, St. Louis, MO 63130, U.S.A.

* Correspondence: mjackrel@wustl.edu

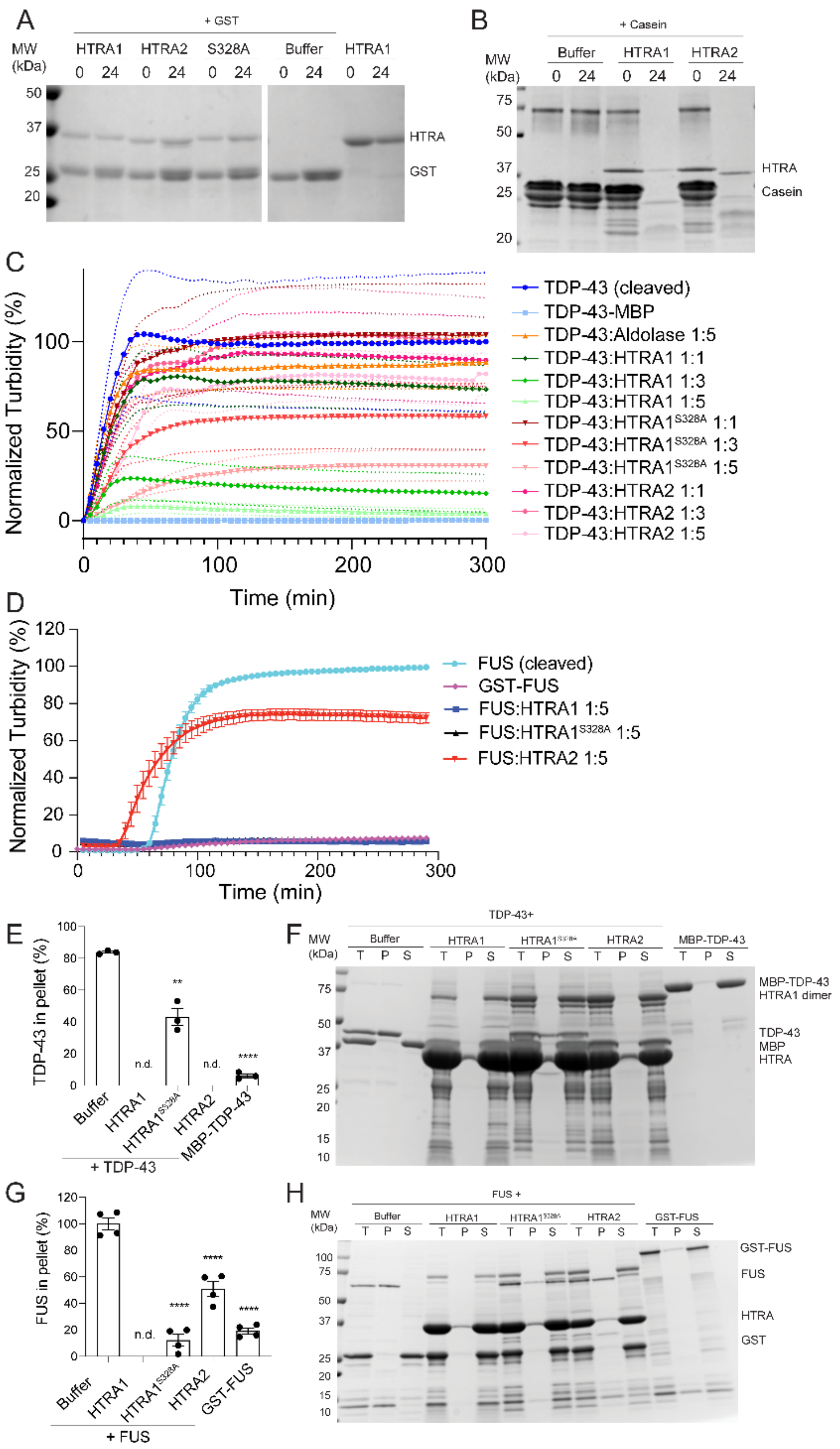

Supplementary Figure 1. HTRA1 does not proteolyze folded substrates such as GST, but does proteolyze casein and can inhibit TDP-43 and FUS aggregation. (A) GST (25μM) was

treated with buffer, HTRA1, or HTRA2 (5μM) for 24h at 37ºC while (B) casein (40μM) was treated with buffer, HTRA1, or HTRA2 (2.5μM) for 24h at 37ºC. Samples were then processed by SDS-PAGE to assess proteolysis (N = 1 (A), N = 3 (B) independent experiments). (C) TDP-43-TEV-MBP (10μM) was incubated with buffer, HTRA1, HTRA1^{S328A}, HTRA2, or aldolase (10-50μM HTRA or 50μM aldolase). Reactions were initiated by addition of TEV protease and aggregation was monitored by turbidity. (N = 3 independent experiments, N = 2 for aldolase condition, means are shown as large symbols, SEM is shown as smaller symbols of the same color). (D) GST-TEV-FUS (5μM) was incubated with buffer, HTRA1, HTRA1^{S328A}, or HTRA2 (25μM). Reactions were initiated by addition of TEV protease at t=0 and aggregation was monitored by turbidity. (N = 4 independent experiments, means are shown as large symbols, SEM is shown as error bars of the same color). (E) TDP-43-TEV-MBP (5μM) was incubated with buffer or the indicated HTRA construct (100μM). Samples from the endpoint of the turbidity assay were then assessed by sedimentation assay and quantified by SDS-PAGE. Values were compared to buffer control using a one-way ANOVA with a Dunnett's multiple comparisons test (N = 3 independent experiments, bars are means ± SEM, **p = 0.0002, ****p<0.0001), n.d. indicates not determined. (F) Representative sedimentation SDS-PAGE gel from E (T = total, P = pellet, S = soluble), N = 3 independent experiments. (G) Samples from the endpoint of D were assessed by sedimentation assay and quantified by SDS-PAGE. Values were compared to buffer control using a one-way ANOVA with a Dunnett's multiple comparisons test (N = 4 independent experiments, bars are means ± SEM, ****p<0.0001), n.d. indicates not detected. (H) Representative sedimentation SDS-PAGE gel from G (T = total, P = pellet, S = soluble), N = 3 independent experiments.

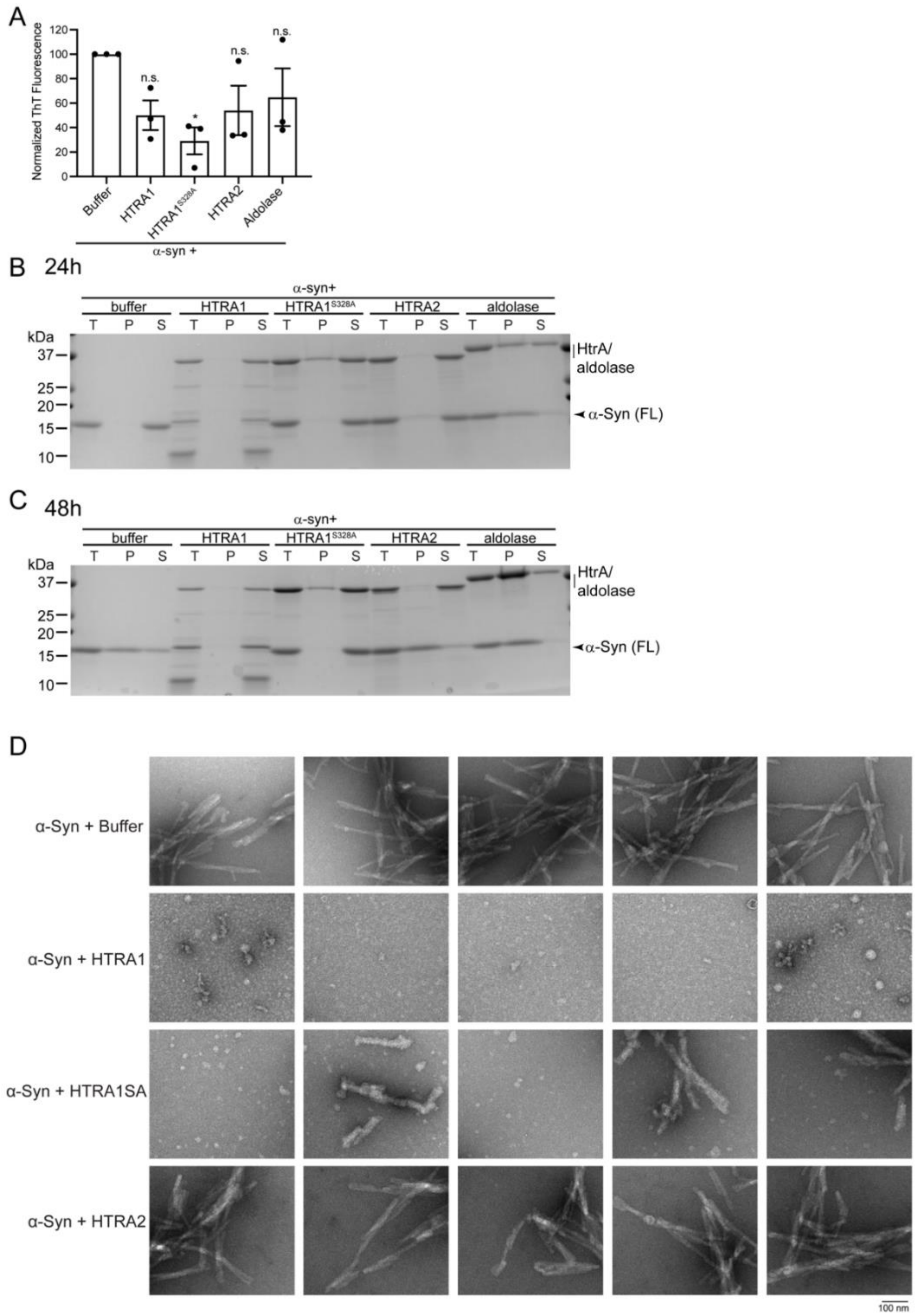

Supplementary Figure 2. HTRA1 prevents α-Syn amyloidogenesis and preserves α-Syn solubility. (A) Experiments were performed as in Fig 2A. Here 72h time point is shown. α-Syn monomer (25 μM) was incubated with buffer, HTRA1, HTRA1^{S328A}, HTRA2, or aldolase (5μM) for 72h. Amyloid content was assessed by ThioflavinT (ThT) fluorescence. Values are compared to buffer treatment using a one-way ANOVA with a Dunnett's multiple comparisons test (N = 3 independent experiments, biological replicates are shown as dots, bars are means ± SEM, *p = 0.038). (B) Experiments were performed as in Fig 2C. Here 24h (B) and 48h (C) time points are shown. α-Syn (25μM) monomer was incubated with HTRA1, HTRA1^{S328A}, HTRA2, or aldolase (5μM) at 37ºC. At the indicated time points, samples were removed and fibrillization was assessed by sedimentation. (T = total, P = pellet, S = soluble). (D) Fibrillization reactions were performed as in Fig 2F and processed for EM, additional images shown.

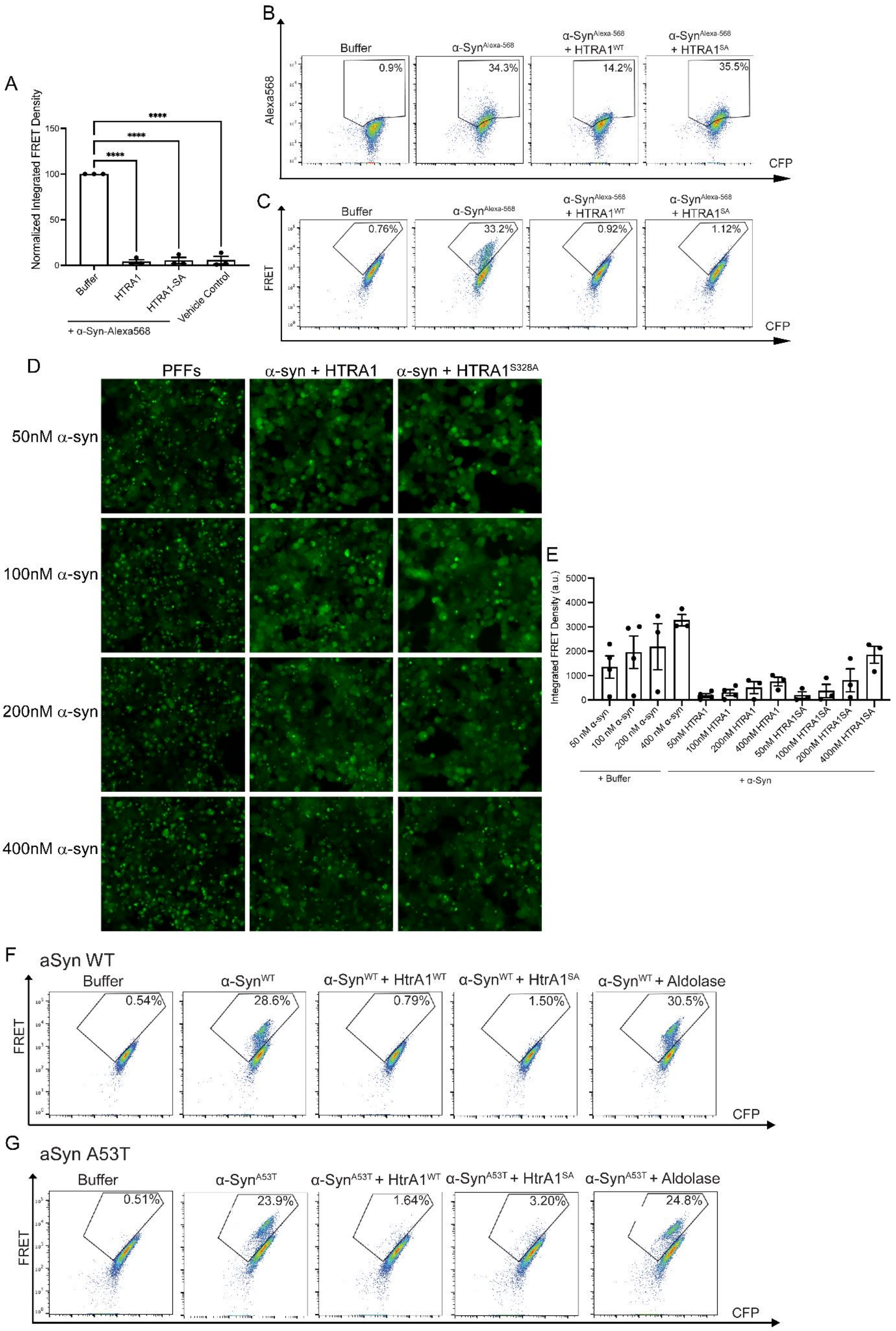

Supplementary Figure 3 HTRA1 prevents α-Syn from forming seeding competent species.

(A) α-Syn-Alexa568 monomer (25μM) was incubated with buffer, HTRA1, or HTRA1^{S328A} (5μM) at 37ºC for 48h. Reaction products were transduced into HEK293T biosensor cells (50nM α-syn in media). Cells were analyzed by flow cytometry 48h following treatment and integrated FRET density was calculated. Values are compared to buffer treatment using a one-way ANOVA with Dunnett's multiple comparisons test (N = 3 independent experiments, ****p<0.0001). (B) Raw flow cytometry data to accompany Fig 3B. (C) Raw flow cytometry data to accompany S3A. (D) Experiments were performed as described in Figure 3B, but with varying concentrations of treated α-syn applied to biosensor cells. Here, α-syn (25μM) monomer was incubated with buffer, HTRA1, or HTRA1^{S328A} (5μM) at 37ºC for 48h. Reaction products were transduced into HEK293T biosensor cells at the indicated concentration of α-syn in media. Cells were assessed by microscopy 48h following treatment. (E) Cells from (D) were analyzed by flow cytometry and integrated FRET density was calculated. (N = 3 for 50nM α-syn + buffer, 100nM α-syn + buffer, and α-syn + HTRA1, N = 3 for all other conditions, biological replicates are shown as dots, bars represent means ± SEM). (F-G) Raw flow cytometry data to accompany Fig 3D-E.

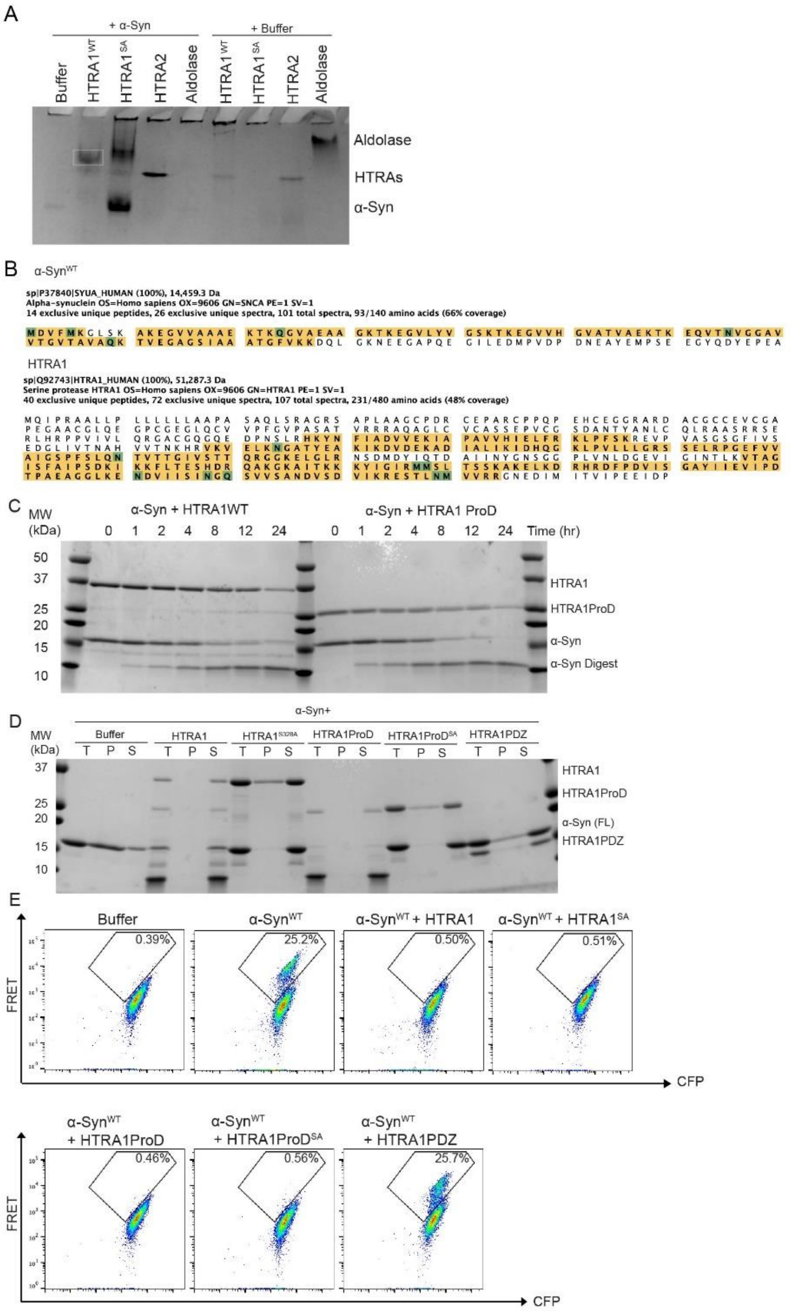

Supplementary Figure 4. The protease domain of HTRA1 is necessary and sufficient for remodeling α-syn. (A) α-Syn (25μM) monomer or buffer was incubated with the indicated HTRA construct. Following shaking for 24h at 37ºC, the reactions were processed for native PAGE. Band outlined in white box was excised and subjected to mass spectrometry analysis. N = 3 independent experiments. (B) α-Synuclein and HTRA1 peptides identified by mass spectrometry from native-PAGE band from A are highlighted in orange. Residues highlighted in green indicate sites of oxidation or deamination. (C) α-Syn monomer (25μM) was treated with HTRA1 or HTRA1ProD (5μM) for 24h at 37ºC. Samples were removed at indicated time-points, and then processed by SDS-PAGE to assess α-syn proteolysis. N = 1. (D) Representative sedimentation assay SDS-PAGE gel to accompany Fig 4E, N = 3 independent experiments. (E) Raw flow cytometry data to accompany Fig 4F.

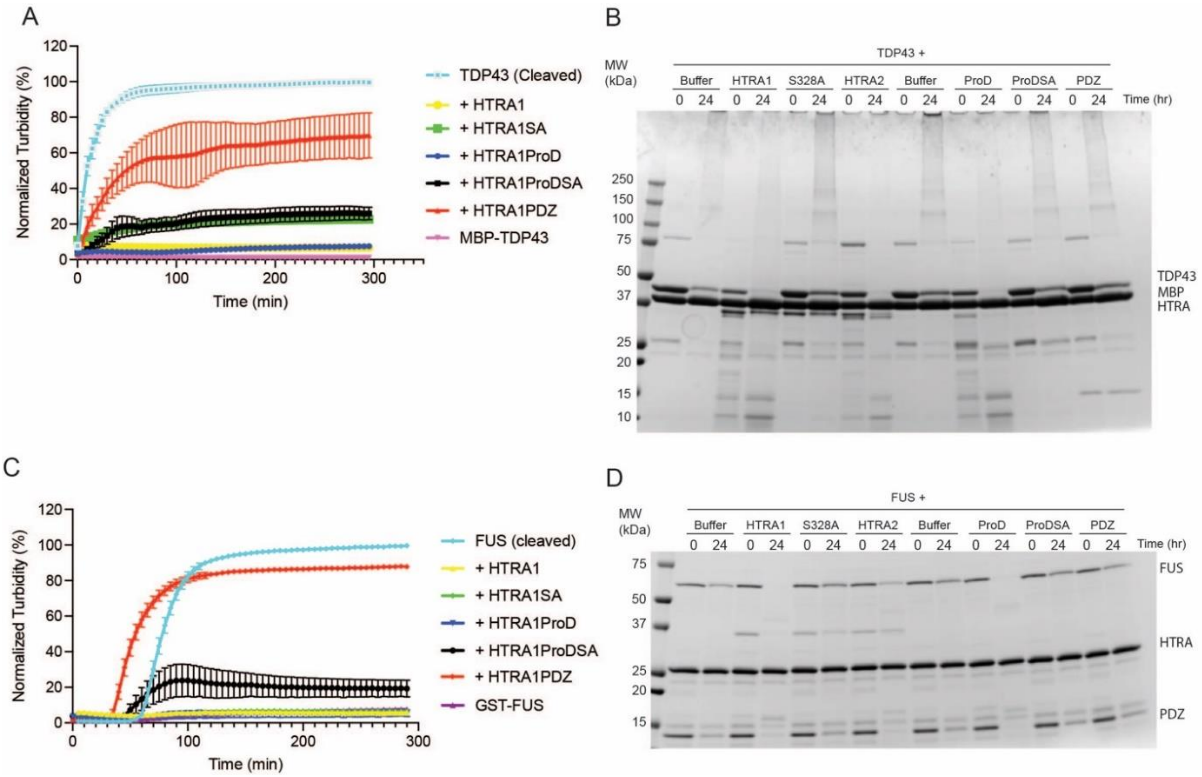

Supplementary Figure 5. Inhibition of TDP-43 and FUS aggregation by HTRA1 is mediated by the protease domain. (A) TDP-43-TEV-MBP (5μM) was incubated with buffer or the indicated HTRA construct (100μM). Reactions were initiated by addition of TEV protease at t=0, and aggregation was monitored by turbidity. (N = 3 independent experiments, means are shown as large symbols, SEM is shown as error bars of the same color). Data for HTRA1 and HTRA1^{S328A} are shown again here for comparison. (B) TDP-43-TEV-MBP (10μM) was treated with TEV protease for 1h at 37ºC, followed by treatment with buffer or the indicated HTRA construct (2μM) for 24h at 37ºC. Samples were then processed by SDS-PAGE. N = 4 independent experiments. (C) GST-TEV-FUS (5μM) was incubated with buffer, HTRA1ProD, HTRA1ProD^{S328A}, or HTRA1PDZ (25μM). Reactions were initiated by addition of TEV protease at t=0 and aggregation was monitored by turbidity. (N = 4 independent experiments, means are shown as large symbols, SEM is shown as error bars of the same color). Data for HTRA1 and HTRA1^{S328A} are shown here again for data comparison. (D) GST-TEV-FUS (10μM) was treated with TEV protease for 1h at 37ºC, followed by treatment with buffer, or indicated HTRA construct (2μM) for 24h at 37ºC. Samples were then processed by SDS-PAGE. N = 4 independent experiments.

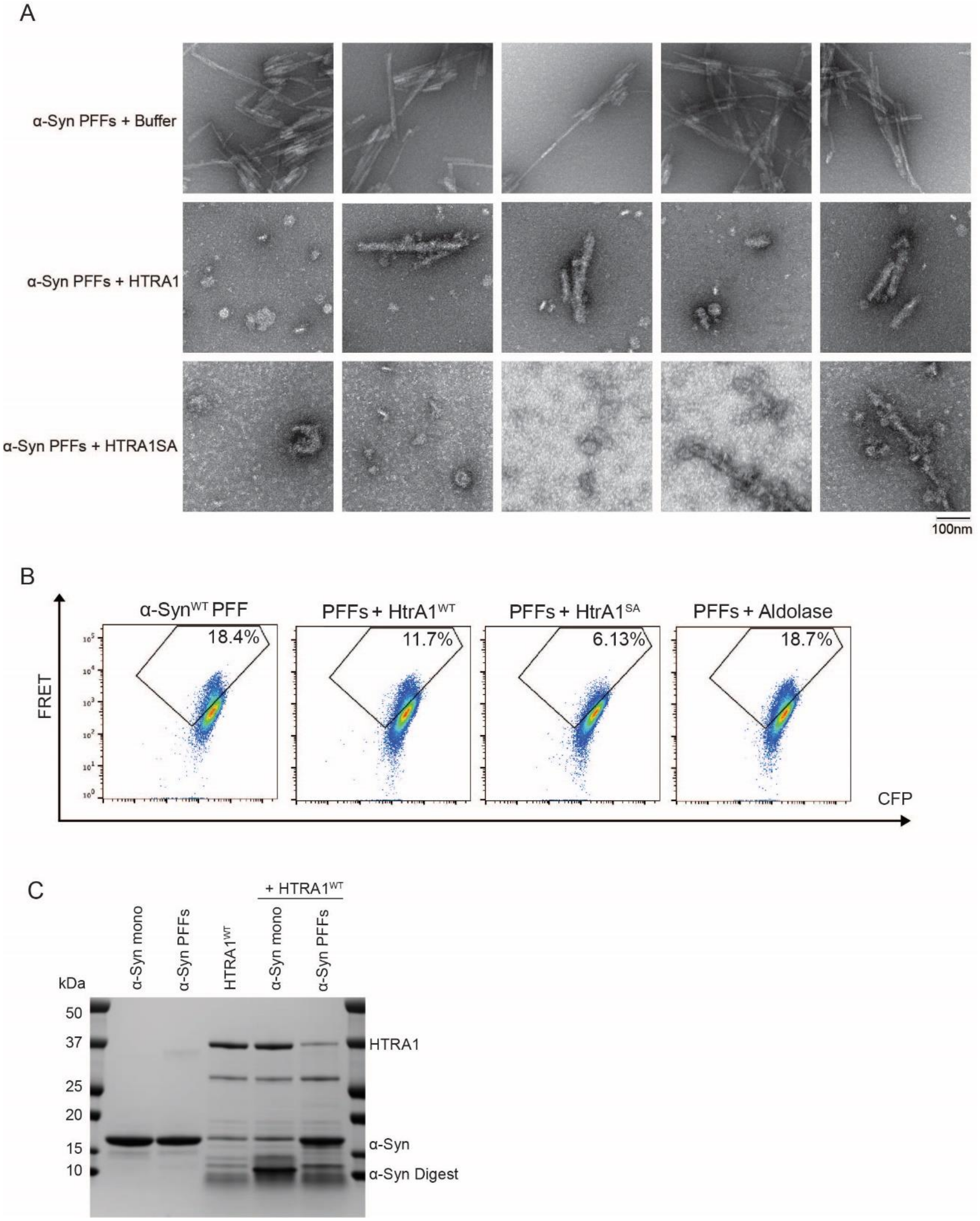

Supplementary Figure 6. HTRA1 treatment remodels α-Syn PFFs to a seeding incompetent form. (A) α-Syn PFFs remodeling reactions were performed as in Fig 5E and processed for EM, additional images shown. (B) Raw flow cytometry data to accompany Fig 5F. (C) α-Syn monomer or fibrils (25μM) was treated with buffer, or HTRA1 (5μM) for 24h at 37ºC. Samples were then processed by SDS-PAGE to assess α-syn proteolysis. N = 2 independent experiments.

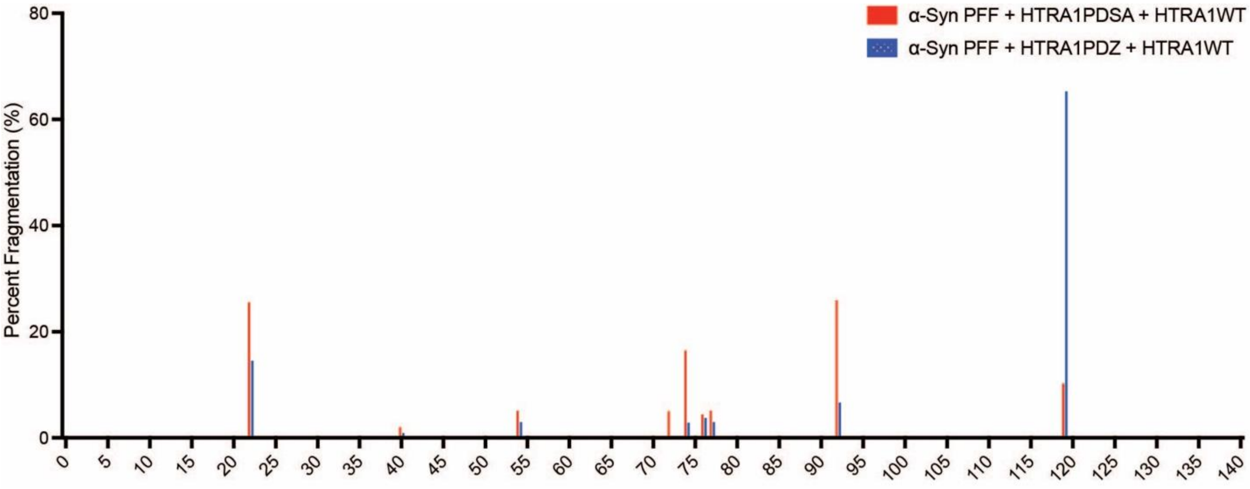

Supplementary Figure 7. HTRA1 disaggregation promotes solubilization and proteolysis of the NAC domain. α-Syn fibrils (5μM) were pre-treated with HTRA1ProD^{S328A} or HTRA1PDZ (50μM) for 2h, followed by addition of HTRA1 (2.5μM) for 3h. Samples were then analyzed by LC/MS. Quantification of the relative abundance of fragmentation at specific cleavage sites is shown. Data for HTRA1ProD^{S328A} pre-treatment is shown here again for data comparison.

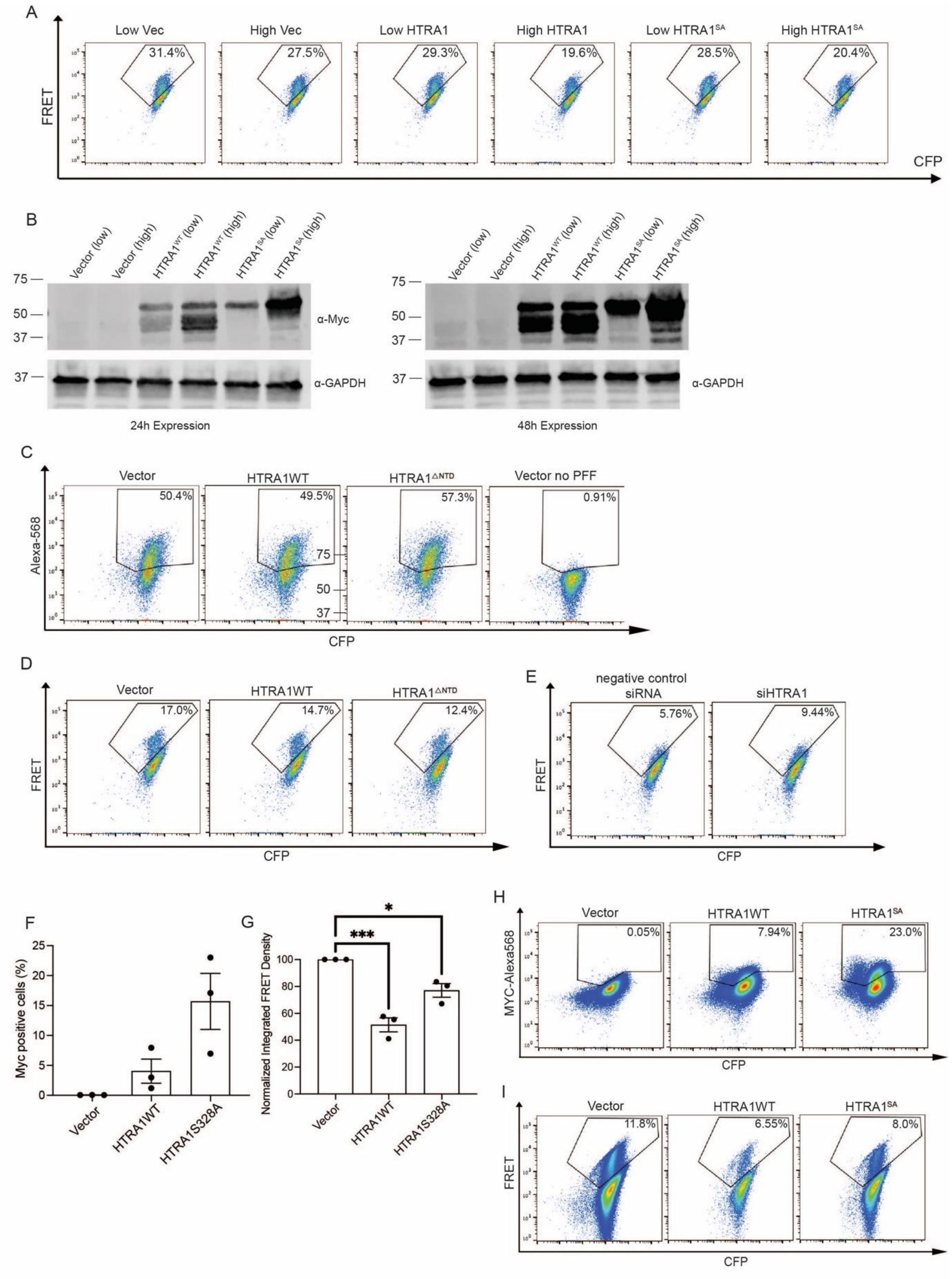

Supplementary Figure 8. Overexpression of HTRA1 prevents α-syn PFFs from seeding aggregation. (A) Raw flow cytometry data to accompany Fig 7B. (B) Experiments were

performed as in Figure 7A-C, with immunoblots performed 24h (left) or 48h (right) post-transfection. N = 3 independent experiments. (C) Raw flow cytometry data to accompany Fig 7D. (D) Raw flow cytometry data to accompany Fig 7E. (E) Raw flow cytometry data to accompany Fig 7H. (F) HEK293T biosensor cells were transfected with HTRA1-Myc, $HTRA1^{S328A}$-Myc plasmid, or a vector control. 2 days following transfection, α-syn PFFs were added to media (50nM). After 1 day of treatment, cells were fixed, immunolabeled with Myc antibody, and analyzed by flow cytometry. Quantification of HTRA1 positive cells is based on Myc signal. N = 3 independent experiments. (G) Quantification of integrated FRET density from F was calculated. (H) Raw flow cytometry data to accompany Fig S8F. (I) Raw flow cytometry data to accompany Fig S8G. Values for all panels were compared to the control expressing vector alone using a one-way ANOVA with a Dunnett's multiple comparisons test (N = 3 independent experiments, biological replicates are shown as dots, bars represent means ± SEM, *p = 0.0145, ***p = 0.0003).

Supplementary Table 1. Oligonucleotides used in this study.

Oligonucleotide	Sequence (5' to 3')
HTRA1_S328A FP	CCATCATCAACTATGGAAAC gcg GGAGGCCCGTTAG
HTRA1_S328A RP	CTAACGGGCCTCC cgc GTTTCCATAGTTGATGATGG
HTRA1_PDZ_Deletion FP	CAGGCCAAAGGAAAAGCCATCACCAAGCTCGAGCACCACCACCACC
HTRA1_PDZ_Deletion RP	CAGTGGTGGTGGTGGTGGTGCTCGAGCTTGGTGATGGCTTTTCCTTTGGC
HTRA1_ProD_Deletion FP	CCCCTCTAGAAATAATTTTGTTTAACTTTAAGAAGGAGATATACCATGAAGAAGTATATTGGTATCCGAATGATGTCACTC
HTRA1_ProD_Deletion RP	GCTGGACGTGAGTGACATCATTCGGATACCAATATACTTCTTCATGGTATATCTCCTTCTTAAAGTTAAACAAAATTATTTC
HTRA1_NTD_Deletion FP	CGGTACCGAGGAGATCTGCCGCCGCGATCGCCATGGGCCAAGGGCAGGAAGATCCC
HTRA1_NTD_Deletion RP	CGGTACCGAGGAGATCTGCCGCCGCGATCGCCATGGGCCAAGGGCAGGAAGATCCC
