# Supplementary material for: HTRA1 disaggregates α-synuclein amyloid fibrils and converts them into non-toxic and seeding incompetent species
Source: Nat Commun. 2024 Mar 18;15:2436. doi: 10.1038/s41467-024-46538-8 (PMC10948756; doi:10.1038/s41467-024-46538-8)
Supplement: Supplementary file 2 — Reporting Summary [file 41467_2024_46538_MOESM2_ESM.pdf]

nature portfolio

Corresponding author(s): Meredith Jackrel

Last updated by author(s): Feb 17, 2024

Reporting Summary

Nature Portfolio wishes to improve the reproducibility of the work that we publish. This form provides structure for consistency and transparency in reporting. For further information on Nature Portfolio policies, see our Editorial Policies and the Editorial Policy Checklist.

Statistics

For all statistical analyses, confirm that the following items are present in the figure legend, table legend, main text, or Methods section.

n/a	Confirmed	
☐	☒	The exact sample size (*n*) for each experimental group/condition, given as a discrete number and unit of measurement
☐	☒	A statement on whether measurements were taken from distinct samples or whether the same sample was measured repeatedly
☐	☒	The statistical test(s) used AND whether they are one- or two-sided<br>*Only common tests should be described solely by name; describe more complex techniques in the Methods section.*
☒	☐	A description of all covariates tested
☐	☒	A description of any assumptions or corrections, such as tests of normality and adjustment for multiple comparisons
☐	☒	A full description of the statistical parameters including central tendency (e.g. means) or other basic estimates (e.g. regression coefficient) AND variation (e.g. standard deviation) or associated estimates of uncertainty (e.g. confidence intervals)
☐	☒	For null hypothesis testing, the test statistic (e.g. *F*, *t*, *r*) with confidence intervals, effect sizes, degrees of freedom and *P* value noted<br>*Give P values as exact values whenever suitable.*
☒	☐	For Bayesian analysis, information on the choice of priors and Markov chain Monte Carlo settings
☒	☐	For hierarchical and complex designs, identification of the appropriate level for tests and full reporting of outcomes
☒	☐	Estimates of effect sizes (e.g. Cohen's *d*, Pearson's *r*), indicating how they were calculated

Our web collection on statistics for biologists contains articles on many of the points above.

Software and code

Policy information about availability of computer code

Data collection	NIS-Elements AR 3.2 was used to acquire HEK cell images.<br>Leica LAS X was used to acquire primary neuron images.<br>Tecan SparkControL was used to collect microplate fluorescence reader data<br>BioTek Gen 5 was used to collect microplate reader data<br>Life Technologies StepOne Software v2.3 was used collect RT-qPCR data<br>Licor Image Studio 5.5.4 was used to acquire western-blot images<br>Bio-Rad Image Lab 6.0.1 was used to acquire gel images
Data analysis	FlowJo V10 was used for flow cytometry analysis<br>Fiji (ImageJ 2.0.0) was used to contrast and overlay images<br>GraphPad Prism 9.3.1 was used for all statistical analysis and graph plotting<br>Li-COR Image Studio Studio Lite was used to contrast western-blot images and for densitometry analysis<br>MaxQuant V2.0.3.0 was used to annotate Mass Spectrometry data<br>Syline-daily V 22.2.1.351 was used to analyze Mass Spectrometry data<br>Image Lab 6.1.0 was used to perform densitometry analysis

For manuscripts utilizing custom algorithms or software that are central to the research but not yet described in published literature, software must be made available to editors and reviewers. We strongly encourage code deposition in a community repository (e.g. GitHub). See the Nature Portfolio guidelines for submitting code & software for further information.

Data

Policy information about availability of data

All manuscripts must include a data availability statement. This statement should provide the following information, where applicable:

- Accession codes, unique identifiers, or web links for publicly available datasets
- A description of any restrictions on data availability
- For clinical datasets or third party data, please ensure that the statement adheres to our policy

The mass spectrometry data generated in this study have been deposited in the ProteomeXchange consortium via the JPOST repository under the accession code: PXD044806 [http://proteomecentral.proteomexchange.org/cgi/GetDataset?ID=PXD044806] (HTRA1 α-Syn native-PAGE experiment) and PXD041784 [http://proteomecentral.proteomexchange.org/cgi/GetDataset?ID=PXD041784] (HTRA1 disaggregation and proteolysis experiment) for ProteomeXchange and JPST002295 and JPST002140 for jPOST. Source data are provided with this paper.

Human research participants

Policy information about studies involving human research participants and Sex and Gender in Research.

Reporting on sex and gender	N/A
Population characteristics	N/A
Recruitment	N/A
Ethics oversight	N/A

Note that full information on the approval of the study protocol must also be provided in the manuscript.

Field-specific reporting

Please select the one below that is the best fit for your research. If you are not sure, read the appropriate sections before making your selection.

[x] Life sciences   [ ] Behavioural & social sciences   [ ] Ecological, evolutionary & environmental sciences

For a reference copy of the document with all sections, see nature.com/documents/nr-reporting-summary-flat.pdf

Life sciences study design

All studies must disclose on these points even when the disclosure is negative.

Sample size	All experiments were performed with at least three biological replicates to ensure reproducibility and allow for statistical analysis. Statistical analysis was performed using Graphpad Prism version 9.3.1 software. All statistical information is described in the figure legends.
Data exclusions	For Mass Spectrometry data analysis low-abundance peptides with peak areas below 10,000 mAU were excluded from the data set. One data point was determined as a statistical outlier using an outlier test in Graphpad, and this particular point was removed from Figure 4d.
Replication	All experimental results were replicated at least three times, and the results were reproducible.
Randomization	Samples were randomized whenever possible. Randomization was not applied to cell culture experiments, randomization was not possible for these in vitro experiments. For imaging experiments, samples were allocated into experimental groups, and were randomly selected within the group. Cells and regions for imaging were selected randomly.
Blinding	Mass Spectrometry experiments and Electron Microscope imaging were performed single-blindly, investigator did not know the identity of the sample prior to data analysis. For other experiments, investigators were not blinded to the identity of the samples, but findings were replicated by multiple investigators.

Reporting for specific materials, systems and methods

We require information from authors about some types of materials, experimental systems and methods used in many studies. Here, indicate whether each material, system or method listed is relevant to your study. If you are not sure if a list item applies to your research, read the appropriate section before selecting a response.

Materials & experimental systems

n/a	Involved in the study
☐	☒ Antibodies
☐	☒ Eukaryotic cell lines
☒	☐ Palaeontology and archaeology
☒	☐ Animals and other organisms
☒	☐ Clinical data
☒	☐ Dual use research of concern

Methods

n/a	Involved in the study
☒	☐ ChIP-seq
☐	☒ Flow cytometry
☒	☐ MRI-based neuroimaging

Antibodies

Antibodies used

All antibodies and associated information listed in Method are also provided below:

Mouse anti-syn (BD Science Cat 610787, at 1:1000 dilution (WB))
Mouse anti-myc (Proteintech Cat 60003-2-IG, at 1:1000 dilution (WB & IF))
Rabbit anti-GAPDH Proteintech, CAT#10494-1-AP, at 1:2500 dilution (WB))
Goat anti-Rabbit IgG LiCor (Cat 926-68071, at 1:2500 dilution (WB))
Goat anti-mouse IgG LiCOR (Cat 926-32210, at 1:2500 dilution (WB))
Rabbit anti-pSyn (Abcam MJFR13, ab168381, at 1:5000 dilution (IF))
Mouse anti-tau (Millipore Sigma, Clone T49, CAT#MABN827, at 1:2000 dilution (IF))
Rabbit anti-myc (Cell Signaling, Cat#2278, at 1:200 (FC), 1:500 (WB) dilutions)
Goat anti-mouse Alexa 568 (Invitrogen, CAT#A-11004, at 1:1000 dilution (IF))
Goat anti-Rabbit Alexa 568 (Invitrogen, CAT#A-11011, at 1:1000 dilution (IF))
Goat anti-Mouse Alexa 488 (Invitrogen, CAT#A-11001, at 1:1000 dilution (IF))

Validation

All antibodies listed above are commercially available and validated by correspondent suppliers, which is described in the manufacturer's website.

https://www.bdbiosciences.com/en-us/products/reagents/microscopy-imaging-reagents/immunofluorescence-reagents/purified-mouse-anti-synuclein.610787
https://www.ptglab.com/products/MYC-Antibody-60003-2-Ig.htm
https://www.ptglab.com/products/GAPDH-Antibody-60004-1-Ig.htm
https://www.licor.com/bio/reagents/irdye-680rd-goat-anti-rabbit-igg-secondary-antibody
https://www.licor.com/bio/reagents/irdye-800cw-goat-anti-mouse-igg-secondary-antibody
https://www.abcam.com/alpha-synuclein-phospho-s129-antibody-mjf-r13-8-8-ab168381.html
https://www.sigmaaldrich.com/US/en/product/mm/mabn827
https://www.cellsignal.com/products/primary-antibodies/myc-tag-71d10-rabbit-mab/2278
https://www.thermofisher.com/antibody/product/Goat-anti-Mouse-IgG-H-L-Secondary-Antibody-Recombinant-Polyclonal/A28175
https://www.thermofisher.com/antibody/product/Goat-anti-Mouse-IgG-H-L-Cross-Adsorbed-Secondary-Antibody-Polyclonal/A-11004
https://www.thermofisher.com/antibody/product/Goat-anti-Rabbit-IgG-H-L-Cross-Adsorbed-Secondary-Antibody-Polyclonal/A-11011

Eukaryotic cell lines

Policy information about cell lines and Sex and Gender in Research

Cell line source(s)

Primary hippocampal neurons from Embryonic-18 CD-1 mice.
HEK293T aSyn biosensor cells were obtained from Tritia Yamasaki.

Authentication

Morphology and characteristics of the cell lines are examined and confirmed with microscopy

Mycoplasma contamination

The cell lines were not tested for Mycoplasma contamination, no appearance or indication of contamination was observed.

Commonly misidentified lines
(See ICLAC register)

There were no commonly misidentified cell lines used in this study

Flow Cytometry

Plots

Confirm that:

☒ The axis labels state the marker and fluorochrome used (e.g. CD4-FITC).

☒ The axis scales are clearly visible. Include numbers along axes only for bottom left plot of group (a 'group' is an analysis of identical markers).

☒ All plots are contour plots with outliers or pseudocolor plots.

☒ A numerical value for number of cells or percentage (with statistics) is provided.

Methodology

Sample preparation	HEK293T biosensor cells were obtained from Tritia Yamasaki. Cells post-treatment were harvested with 0.05 % Trypsin. Cells were pelleted and resuspended in 4% paraformaldehyde for fixation. Cells were then pelleted again and immunostained, or resuspended in MACSQuant Flow Running buffer for analysis.
Instrument	MACSQuant VYB
Software	FlowJo 10.5.3
Cell population abundance	At least 10,000 single cells were analyzed for each experiment
Gating strategy	SSC-A/FSC-A gate was drawn to select intact cells after paraformaldehyde fixation. Then FSC-H/FSC-A gate was drawn to define single cells and exclude cell doublets. In FRET-A and CFP-A bivariate plot, a polygon gate was drawn in control cells such that the FRET-negative and -positive populations are separated.

☒ Tick this box to confirm that a figure exemplifying the gating strategy is provided in the Supplementary Information.
